# *mSystems*: Learning To Love Systems

**DOI:** 10.1128/mSystems.00015-15

**Published:** 2015-12-22

**Authors:** Jack A. Gilbert

**Affiliations:** Argonne National Laboratory, Biosciences Division, Lemont, Illinois, USA; Department of Surgery, University of Chicago, Chicago, Illinois, USA; Marine Biological Laboratory, Woods Hole, Massachusetts, USA

## Abstract

We want this journal to be a forum for publication of research, tools, and data sets that are advancing our understanding of, or ability to investigate or interpret, microbiology systems. For example, I hope that *mSystems* will be a venue for research that elucidates the metabolic and cell-cell signaling mechanisms that underpin observed successional dynamics in microbial ecosystems.

## EDITORIAL

So what actually is systems biology? This is probably a good question to ask, especially considering that the ASM is investing in publishing an entire journal devoted to it. Well, microbial systems biology, for the purposes of this journal, is inclusive of all research exploring the relationships between the components of a microbial cell or community. Understanding systems biology is an increasingly important consideration in the current multi-omic methods of microbiological investigation. A system is a set of connected components forming a complex whole. A systems component, in a microbiological context, can comprise any biological or even physicochemical element that connects to produce the observed characteristics of the system. These connections can be at any level in the system, can span levels, and can lead to explainable characteristics as well as emergent properties that are less predictable based on our current understanding of the system. This does sound rather comprehensive, especially to a microbial ecologist like me, but we feel that this represents a unique discipline.

We want this journal to be a forum for publication of research, tools, and data sets that are advancing our understanding of, or ability to investigate or interpret, microbiology systems. For example, I hope that *mSystems* will be a venue for research that elucidates the metabolic and cell-cell signaling mechanisms that underpin observed successional dynamics in microbial ecosystems. I love time series, and therefore, exploring how these communities colonize an environment and then change through time, both through interaction within and between species and through constraints based on the physicochemical dynamics of the environment, is particularly interesting to me—and to many others, I am sure. I asked members of our Senior Editorial Board what they hoped to see published in *mSystems*. Margaret McFall Ngai said that she hopes “*mSystems* will be a venue for the announcement of new, breakthrough molecular and cellular technologies associated with understanding structure and function of microbial communities. Such technologies will come not only from the microbiology community, but through the collaboration of these scientists with engineers and colleagues in the physical sciences.” Jeroen Raes is interested in “*mSystems* publications that can help untangle the wiring of ecosystem species interactions to open the way toward targeted modulation strategies.” Julie Huber said that “by examining microbes at multiple scales across multiple ecosystems,” she hopes *mSystems*’ publications “will begin to formulate what common rules, if any, govern the assembly of microbial phylogenetic, genomic, and functional organization.” With our broad range of editors, we are set to be able to provide peer review for any level of organisms in microbial systems, from the cell to the population and from the community to the ecosystem.

In a world where researchers can collaborate across many disciplines in order to advance research, microbial systems biology requires integration of data types and the development of novel laboratory platforms and bioinformatics tools that can advance research. We are pleased to have this venue where we as a community can present our most impactful research in a fully open-access (CC-BY) forum. We will work hard to ensure that *mSystems* is the place people turn to when they want to find the cutting-edge studies being published in this field.

